# Assessing the uniqueness of language: Animal grammatical abilities take center stage

**DOI:** 10.3758/s13423-016-1091-9

**Published:** 2016-07-01

**Authors:** Carel ten Cate

**Affiliations:** Behavioural Biology, Institute of Biology Leiden and Leiden Institute for Brain and Cognition, Leiden University, P.O. Box 9505, 2300 RA Leiden, The Netherlands

**Keywords:** Language evolution, Artificial grammar learning, Comparative cognition, Animal cognition, Animal vocalizations

## Abstract

Questions related to the uniqueness of language can only be addressed properly by referring to sound knowledge of the relevant cognitive abilities of nonhuman animals. A key question concerns the nature and extent of animal rule-learning abilities. I discuss two approaches used to assess these abilities. One is comparing the structures of animal vocalizations to linguistic ones, and another is addressing the grammatical rule- and pattern-learning abilities of animals through experiments using artificial grammars. Neither of these approaches has so far provided unambiguous evidence of advanced animal abilities. However, when we consider how animal vocalizations are analyzed, the types of stimuli and tasks that are used in artificial grammar learning experiments, the limited number of species examined, and the groups to which these belong, I argue that the currently available evidence is insufficient to arrive at firm conclusions concerning the limitations of animal grammatical abilities. As a consequence, the gap between human linguistic rule-learning abilities and those of nonhuman animals may be smaller and less clear than is currently assumed. This means that it is still an open question whether a difference in the rule-learning and rule abstraction abilities between animals and humans played the key role in the evolution of language.

The complexity of human language structure has given rise to fundamental questions regarding the nature and evolutionary origin of this complexity: To what extent does language structure deviate from the vocal communication signals of nonhuman animals? Are the computational and learning mechanisms that guide learning about language structure specific to language or to humans? These questions have in common that addressing them requires adequate knowledge of the relevant abilities of nonhuman animals. Studies on these abilities have used various approaches. One is to focus on the production of species-specific vocalizations, to compare the syntax of animal vocalizations with that of language. Another is to focus on perceptual and processing abilities more generally, through experiments on “artificial grammar learning” (AGL) that assess what types of patterns or grammar rules animals can detect in artificially prepared string sets. The value of comparative studies for shedding light on the uniqueness and possible origins of language structure is widely acknowledged, but I will argue that we are still only scratching the surface of the relevant cognitive abilities of animals.

## The structure of animal vocalizations

The vocalizations of the closest living relatives of humans, the great apes, are quite unlike those of humans. Their repertoire is limited and does not result from vocal learning, and most vocalizations do not consist of longer sequences of discrete sounds. So, if one is interested in understanding how the structural complexity of language may have arisen, studying great ape vocalizations does not provide the most useful model. However, other animal species have rich, learned, and more varied vocalizations—in particular, songbirds and cetaceans (dolphins and whales). Their variability has made these vocalizations a prime target for formal analyses of their underlying structures, often using the Chomsky hierarchy as a yardstick to measure the complexity of animal vocalizations (e.g., Berwick, Okanoya, Beckers, & Bolhuis, 2011). This has revealed that although the songs of a nightingale (*Luscinia megarhynchos*) or a humpback whale (*Megaptera novaeangliae*) are more complex than the first- or second-order Markov chains with which the first researchers described birdsong structures (reviewed by ten Cate, Lachlan, & Zuidema, 2013), the structure of many animal vocalizations can be described by such algorithms as probabilistic or hidden Markov models (Berwick et al., 2011; Hurford, 2012; ten Cate et al., 2013; ten Cate & Okanoya, 2012), “state chain” processes (Hurford, 2012), or “renewal processes” (Kershenbaum et al., 2014). However, all of these processes belong to a lower level of the hierarchy than the context-free grammars needed for a formal description that could produce the recursive patterns characteristic of human language (Berwick et al., 2011; Hurford, 2012). One may draw the conclusion that this sufficiently demonstrates the presence of a fundamental gap between language and animal vocalizations, but this conclusion may be drawn too quickly. To fully capture the features of complex vocalizations such as the song of a nightingale, with different levels of organization (elements within subsections of songs; subsections within a song; and songs within a string of songs), may require models that include one or more submodels describing the organization of a different, lower level, creating a hierarchical structure. Several authors have suggested that such models may reduce the gap between human and animal linguistic abilities (Hurford, 2012; Kershenbaum et al., 2014; ten Cate et al., 2013). Also, although they may not capture the complexity present in lexical syntax, they might have a level of complexity comparable to models for human morphosyntax (Samuels, 2015). At the same time, the vocalizations of many species still need to be explored. In doing so, we may discover species with vocalizations that will further narrow the current gap.

## Language as cognitive process

Although studying the structural regularities in the vocalizations of nonhuman animals can be informative, we may question whether this could ever yield the full or most useful insights into the roots of linguistic structures. To understand why, we should look at the lively debate concerning the uniqueness of language. On one side of the spectrum, we find those who argue that human language is based on the evolution of a special, innate language faculty, consisting of some uniquely human computational abilities (“universal grammar”)—in particular, the use of recursive structures (e.g., Everaert, Huybregts, Chomsky, Berwick, & Bolhuis, 2015). Proponents of this view argue that language did not evolve for communication, but instead reflects advanced and uniquely human cognitive abilities that evolved primarily as instruments for thought and combining information (e.g., Berwick & Chomsky, 2015). On the other side of the spectrum, we find those who argue that language is “usage based” (Tomasello, 2003) and evolved from domain- and species-general computational mechanisms, with humans having more advanced computational mechanisms (e.g., Christiansen & Chater, 2015). Proponents of this view also argue that these cognitive mechanisms evolved prior to language and subsequently made language possible. These opposing views concerning the uniqueness of the mechanisms underlying language development and their evolution both refer to animal studies to support their cases, either by arguing that the current animal studies show no evidence of the cognitive mechanisms required for linguistic syntax (e.g., Watumull, Hauser, & Berwick, 2014) or by arguing that the cognitive mechanisms of nonhuman animals provided the basis for the more advanced ones present in humans (e.g., Christiansen & Chater, 2015). Thus, if the roots of language are to be found in cognitive mechanisms that did not primarily evolve for communication, we should look beyond the structure or processing of species-specific vocalizations. Instead, the computational and rule-learning abilities of nonhuman animals in general, and how these compare to those of humans, take center stage in the debate about the uniqueness of human language. So, what do we know about these topics?

## Animal artificial grammar learning

If human computational abilities have their origin outside the domain of language, there is no need to restrict comparative studies to animal species that show elaborate vocalizations or vocal learning. Also, studying how visual patterns are processed may be equally as informative, in terms of the abilities for pattern detection and rule learning, as studying auditory processing. Such studies have demonstrated that processes like categorization and concept formation, which are also essential for language processing, can be found in a range of nonhuman animals (e.g., Zentall & Wasserman, 2012). Comparative studies have also demonstrated that various animals can use ordinal as well as transitional information to learn about linear strings of items (e.g., Chen & ten Cate, 2015; Orlov, Amit, Yakovlev, Zohary, & Hochstein, 2006), and that some species can detect nonadjacent relations among items (e.g., Sonnweber, Ravignani, & Fitch, 2015).

The abilities to process more abstract or higher-order relationships among items are more explicitly tested in AGL experiments, in which arbitrary, meaningless auditory or visual items are presented to subjects in strings arranged according to particular grammatical rules that define the sequences of, or the relations between, particular items. Next, subjects are tested for whether and what they learn about the underlying structures. This method has proven very successful for analyzing and understanding the rule-learning mechanisms and their constraints in human adults and infants, and increasingly it is being used to address the sequence-learning abilities of nonhuman animals (Fitch & Friederici, 2012). Studies of infants are often based on a familiarization paradigm, in which they are exposed to a string of sounds organized according to one particular algorithm. After hearing this for some time, resulting in a decline of interest in the sounds, the infants hear test sounds organized according to either the familiarized or a deviating structure. A difference in attending to the familiar and deviant-structured strings (measured by behavioral or neural responses) indicates that the infants have detected a difference between the two structures (e.g., Marcus, Vijayan, Rao, & Vishton, 1999). The familiarization method has been and is being used to test several animal species, in particular various monkey species (e.g., Hauser & Glynn, 2009). A second frequently used method, especially for animal testing, is to use operant conditioning to train animals to discriminate sets of strings organized according to a different algorithm. Making the distinction already indicates that the animals are able to detect a difference in structure. But, as has been noted for rule learning in humans (e.g., Aslin & Newport, 2014; Gerken, 2006; Kovacs, 2014), sets of strings can be discriminated in many ways, ranging from rote memorization of different strings to deriving the abstract rule underlying a set. Subsequent probe trials using novel, nonreinforced, differently structured strings are needed to test various hypotheses as to what exactly has been learned about the training sets (e.g., Ravignani, Westphal-Fitch, Aust, Schlumpp, & Fitch, 2015; van Heijningen, Chen, van Laatum, van der Hulst, & ten Cate, 2013). Both familiarization and discrimination tasks have their pros and cons (see ten Cate & Okanoya, 2012, for a discussion) but can be used to address similar questions. Because both types of studies can provide complementary information, combining them might be useful, but this is something still to be exploited.

In principle, AGL experiments on rats and vocal-nonlearning birds like pigeons can be just as informative, regarding animal rule-learning abilities, as those focused on primates or vocal-learning songbirds, as several studies have demonstrated. Pigeons (*Columba livia*), for instance, could learn to discriminate two different artificial finite state grammars consisting of strings of colored letters, and could generalize this discrimination to novel strings (Herbranson & Shimp, 2008), and rats (*Rattus norvegicus*) could detect regularities in a task in which they had to press levers in a hierarchically organized sequence (Fountain et al., 2012). However, the elaborate vocal patterns in the songs of many songbirds and their abilities to learn such patterns might make this group a particularly promising one to find more elaborate abilities for processing and learning of auditory patterns. If one’s interest is in an animal model as a comparison to study the neural mechanisms underlying such processing, then primates may provide the best entry. For instance, Wilson et al. (2015) used fMRI to examine the brain regions involved in processing a forward-branching artificial finite state grammar in both rhesus macaques (*Macaca mulatta*) and humans. They found counterparts in the monkey brain for regions in both the human ventral frontal and opercular cortices that are associated with the initial stages of human syntax processing. These results hint at the possibility that regions that currently play a significant role in language function may have had their origin in more domain-general sequence-processing regions that may have also been present in our ancestors (Wilson et al., 2015).

## Can animals learn rules?

Several AGL studies have addressed whether animals can learn more abstract rules, such as detecting the regularity in strings of arbitrary sound items arranged in an (AB)^n^ or A^n^B^n^ pattern (A and B indicating sounds belonging to two different categories). The second pattern requires the animal to keep track of the number of As to assess whether the proper number of B items is present, which is not required for keeping track of the AB alternation in the first pattern and crosses the border of what a finite state grammar can deal with. Whereas humans succeeded in detecting both patterns, tamarins (*Saguinis oedipus*) could only detect the first one (Fitch & Hauser, 2004). Subsequent studies on starlings (*Sturnus vulgaris*; Gentner, Fenn, Margoliash, & Nusbaum, 2006) and zebra finches (*Taeniopgia guttata*; van Heijningen, de Visser, Zuidema, & ten Cate, 2009) showed that both species were able to distinguish (AB)^n^ from A^n^B^n^ strings. Although this might suggest that they had learned the rules underlying the string sets, demonstrating true rule abstraction would require two conditions to be met: (1) maintaining the distinction when sounds belonging to other categories than the A and B training categories were used, and (2) correct classification of probe strings with items arranged in sequences that either did or did not fit the specific algorithms of the training strings. Further tests of the zebra finches showed that only one individual transferred the distinction to sounds from novel categories (C and D). This suggests that, for most birds, the original discrimination might have been based on generalization of specific phonetic features of the A and B items, rather than on abstracting the pattern. In addition, probe tests with other string types revealed that the birds discriminated the strings by attending to local regularities in the strings, such as the presence of AA bigrams (which are only present in A^n^B^n^ strings), rather than by attending to the overall structure. These discrimination strategies differed between individuals (van Heijningen et al., 2009), demonstrating the importance of analyzing the responses to probe strings at the individual level. The starlings were not tested with sound items belonging to novel categories, nor did the researchers examine how individual birds discriminated the strings. So, although birds may have the ability to detect higher-order string regularities, this was not unambiguously demonstrated by these experiments. Similar experiments with pigeons and keas (*Nestor notabilis*), using strings of visual items, showed that these species also based discriminations between the two string types on the presence or absence of local regularities, rather than on the global string structure. Interestingly, the keas all used the same feature to discriminate between the string types (attending to the presence of a BA transition), whereas the pigeons showed individual differences and no consistent pattern in which features they used (Ravignani et al., 2015; Stobbe, Westphal-Fitch, Aust, & Fitch, 2012).

Another series of experiments were inspired by a seminal study by Marcus et al. (1999), in which 7-month-old infants were habituated to XYX or XYY strings (with X and Y being speech syllables, resulting in strings like “gatiga” or “gatiti”). Afterward, the infants’ responses to novel strings showed that they had extracted the underlying regularity and generalized it to stimuli composed of novel syllables. The seemingly simple task of discriminating XYX and XXY structures lends itself very well to comparing how humans and various animal species do this. A number of species have been tested on their abilities to detect this pattern, in both habituation and discrimination tasks, including rhesus macaques (Hauser & Glynn, 2009), rats (Murphy, Mondragón, & Murphy, 2008), Bengalese finches (*Lonchura striata domestica*; Seki, Suzuki, Osawa, & Okanoya, 2013), zebra finches (Chen, van Rossum, & ten Cate, 2015; Spierings & ten Cate, 2016; van Heijningen et al., 2013), and budgerigars (*Melopsittacus undulates*; Spierings & ten Cate, 2016). As with the previous example, these species also distinguished the string types. Some studies claimed to have demonstrated rule abstraction by showing that novel stimuli were responded to appropriately (e.g., Murphy et al., 2008). But here, too, the claims for rule learning have been contested by suggesting that various results can be explained by lower-level similarities between the training and test strings (e.g., Corballis, 2009). So, the questions above can be raised here as well: Is there transfer of the discrimination to sounds that do not match the training sounds phonologically, and what “rule” do the animals use (see also ten Cate, 2014; ten Cate & Okanoya, 2012)?

The ability to transfer a discrimination to strings consisting of novel sounds has been tested systematically in one recent experiment. Both zebra finches and budgerigars were first trained to discriminate a set of XYX from XXY strings, and next were tested with strings consisting of novel arrangements of familiar items and strings consisting of items belonging to novel sound categories that had no acoustical similarity to the training sounds, but only shared the string structure (Spierings & ten Cate, 2016). The results were remarkable. The zebra finches did not differentiate between the XYX and XXY strings consisting of novel items. However, strings consisting of novel arrangements of the familiar items were differentiated, albeit not by their underlying structure, but by the ordinal positions of the individual items. The zebra finches seemed to have memorized the positions of the separate items in all training stimuli and judged new strings by their similarity to the training strings with respect to item position. Earlier experiments had also indicated that zebra finches attend to specific parts of training strings and use these to classify novel arrangements of stimuli (Chen et al., 2015; van Heijningen et al., 2013). In contrast, budgerigars exposed to the same strings behaved quite differently. They did attend to the abstract structural similarity of the training and test strings, similar to the infants in the experiments of Marcus et al. (1999). Whether the difference between the two bird species is related to other cognitive abilities awaits further investigation. In any case, although these species showed a fundamental difference in the strategies they used to discriminate the training stimuli, both strategies were effective for discriminating the training stimuli correctly.

## An unbridgeable gap?

Interestingly, whereas studies using XYX and XXY strings have shown that human infants and adults readily generalize to phonetically novel items, there is also evidence that if the stimulus sets used can be distinguished using local pattern similarities, humans prefer to do so. Infants exposed to XXY strings in which the Y items were the same for all training strings used this Y item as a cue to classify the strings, and used the overall structural pattern only when no such similarity was present (Gerken, 2006). Also, both human infants (Kovacs, 2014) and adults (Chen et al., 2015) are more inclined to classify strings on the basis of the presence of item repetition (e.g., XX), if possible, than to base comparisons on all three items. So, in contrast to what seems to be a common assumption when comparing human and animal computational abilities, and despite the presence of human “dendrophilia” (a sensitivity to higher-order structure; Fitch, 2014), humans do not always use higher-order regularity as a default strategy; sometimes they also rely on lower-level or specific local regularities, similar to the strategies used by nonhuman animals. And if humans can apply different strategies, depending on the context, why not animals? Although animal species may have attended to lower-level regularities as a default, in some cases they may have been able to use higher-level ones, if necessary. Hence, the gap between the inferred default for humans to use higher-order strategies and for animals to use lower ones may be smaller than is sometimes claimed (e.g., Watumull et al., 2014).

To summarize, it is fair to say that the evidence for several claims of animals being able to attend to higher-order regularities in strings, ranging from baboons (*Papio papio*; Rey, Perruchet, & Fagot, 2012) and rats (Murphy et al., 2008), to starlings (Gentner et al., 2006) and Bengalese finches (Abe & Watanabe, 2011), is at best ambiguous (e.g., Beckers, Bolhuis, Okanoya, & Berwick, 2012; Corballis, 2009; Poletiek, Fitz, & Bocanegra, 2016; van Heijningen et al., 2009). Nevertheless, although the findings can be explained by the use of simpler strategies, this does not imply that animals are unable to use higher-order ones. Animals’ real potential for rule learning may not have been revealed by the current experiments; the challenge for researchers will be to create better experiments to test these abilities. At the same time, it is clear that species (and also individuals) tested with the same stimuli can differ in their default strategies for assessing the regularities among strings. This was shown by the pigeons and keas tested by Ravignani et al. (2015), and by the zebra finches and budgerigars discussed above (Spierings & ten Cate, 2016). Because only a handful of species have been tested in AGL experiments, research needs to expand to more species, to explore the strategies with which they approach various tasks, how these compare to human strategies in the same task, and how and why this variation arises. Among bird species, particularly interesting candidates for such studies are corvids (songbirds) and large parrots such as Amazon parrots. Both groups have shown cognitive abilities that seem beyond those of other bird species in several domains, such as tool use (e.g., Auersperg et al., 2014; Taylor, Hunt, Holzhaider, & Gray, 2007), analogical reasoning (e.g., Obozova, Smirnova, Zorina, & Wasserman, 2015; Smirnova, Zorina, Obozova, & Wasserman, 2015), and detecting rhythmic patterns in auditory stimuli (Schachner, Brady, Pepperberg, & Hauser, 2009; ten Cate, Spierings, Hubert, & Honing, 2016), all features that require abilities for abstraction and higher-order pattern recognition. No representatives of these groups so far have been tested with artificial grammar tasks. If it turns out that these or other species do have rule-learning and abstraction abilities that are qualitatively similar to those in humans, it would lend support to the hypothesis that the uniqueness of language is not due to a single, uniquely human processing mechanism. Instead, language’s uniqueness might lie in its combination of quantitative differences, such as a considerable quantitative difference in rule-learning abilities, and the linking of rule-learning and processing mechanisms to a sensory–motor interface for vocal production and to a system providing meanings (semantics; Hurford, 2012).

## Conclusion

To conclude, whether we consider the structure of animal vocalizations or the animal abilities for rule learning, we have only just begun to explore the relevant cognitive abilities and to examine to what extent they differ between humans and animals. There may still be much to discover before we can draw firm conclusions regarding the uniqueness of language.
